# Direct visualization of electroporation-assisted in vivo gene delivery to tumors using intravital microscopy – spatial and time dependent distribution

**DOI:** 10.1186/1471-2407-4-81

**Published:** 2004-11-16

**Authors:** Maja Cemazar, Ian Wilson, Gabi U Dachs, Gillian M Tozer, Gregor Sersa

**Affiliations:** 1 Department of Experimental Oncology, Institute of Oncology, Zaloska 2, SI-1000 Ljubljana, Slovenia; 2 Tumour Microcirculation Group, Gray Cancer Institute, PO Box 100, Northwood HA6 2JR, UK; 3 Angiogenesis Research Group, Department of Pathology, University of Otago, Christchurch, New Zealand; 4 Academic Unit of Surgical Oncology, Division of Clinical Sciences, University of Sheffield, Floor K, Royal Hallamshire Hospital, Sheffield, S10 2JF, UK

## Abstract

**Background:**

Electroporation is currently receiving much attention as a way to increase drug and DNA delivery. Recent studies demonstrated the feasibility of electrogene therapy using a range of therapeutic genes for the treatment of experimental tumors. However, the transfection efficiency of electroporation-assisted DNA delivery is still low compared to viral methods and there is a clear need to optimize this approach. In order to optimize treatment, knowledge about spatial and time dependency of gene expression following delivery is of utmost importance in order to improve gene delivery. Intravital microscopy of tumors growing in dorsal skin fold window chambers is a useful method for monitoring gene transfection, since it allows non-invasive dynamic monitoring of gene expression in tumors in a live animal.

**Methods:**

Intravital microscopy was used to monitor real time spatial distribution of the green fluorescent protein (GFP) and time dependence of transfection efficiency in syngeneic P22 rat tumor model. DNA alone, liposome-DNA complexes and electroporation-assisted DNA delivery using two different sets of electric pulse parameters were compared.

**Results:**

Electroporation-assisted DNA delivery using 8 pulses, 600 V/cm, 5 ms, 1 Hz was superior to other methods and resulted in 22% increase in fluorescence intensity in the tumors up to 6 days post-transfection, compared to the non-transfected area in granulation tissue. Functional GFP was detected within 5 h after transfection. Cells expressing GFP were detected throughout the tumor, but not in the surrounding tissue that was not exposed to electric pulses.

**Conclusions:**

Intravital microscopy was demonstrated to be a suitable method for monitoring time and spatial distribution of gene expression in experimental tumors and provided evidence that electroporation-assisted gene delivery using 8 pulses, 600 V/cm, 5 ms, 1 Hz is an effective method, resulting in early onset and homogenous distribution of gene expression in the syngeneic P22 rat tumor model.

## Background

Despite some promising early results, gene therapy does not, as yet, live up to expectation [1]. The main stumbling block remains gene delivery, and all advances in the control of gene expression and selection of therapeutic genes are hampered by inefficient gene transfection. Hence the development of a safe and effective method of gene delivery *in vivo *is of utmost importance if gene therapy is to move from the experimental to the clinical stage.

Electroporation is currently receiving much attention as a way to increase drug and DNA delivery [2-5]. Electroporation has long been used as an effective *in vitro *gene delivery system in both prokaryotes and eukaryotic cells. Electroporation is a physical means of importing small molecules and macromolecules into cells via increased cell membrane permeability. Electroporation combined with chemotherapeutic drugs bleomycin and cisplatin (electrochemotherapy) has shown to be very promising antitumor therapy. It was tested on many different tumor types on the preclinical level demonstrating high antitumor effectiveness resulting in tumor cures at very low chemotherapeutic doses. It was also tested in clinical trials for the treatment of accessible cutaneous tumors of different histological types in cancer patients resulting in up to 100% objective responses [4,6,7]. Recent studies demonstrated the feasibility of electrogene therapy using a range of therapeutic genes for the treatment of experimental tumors [4,8-14]. However, the transfection efficiency of electroporation-assisted DNA delivery is still low compared to viral methods and there is a clear need to optimize this approach [5,14,15]. In studies designed to determine transfection efficiency in tumors, tissue homogenates, tissue sections or measurement of fluorescence in whole-tumor specimens using fluorescence stereomicroscope have been employed. Different plasmids have also been used to analyze transfection efficiencies, such as those encoding the green fluorescence protein (GFP), β-galactosidase or luciferase [5,14,15], making direct comparisons difficult.

In order to optimize treatment, knowledge about spatial and time dependency of gene expression following delivery is of utmost importance in order to improve gene delivery. This information is also important for the timing of gene therapy with other cancer treatment modalities. Intravital microscopy of tumors growing in dorsal skin fold window chambers is a useful method for monitoring gene transfection, since it allows non-invasive dynamic monitoring of gene expression in tumors in a live animal [16,17]. So far, only one study has used this technique to monitor the activity of an adenovirus in infecting a mammary cell carcinoma over the course of several days [16]. In the present study, we used intravital microscopy to monitor real time spatial distribution of gene transfection using the GFP reporter gene in rat P22 tumors. We followed time dependency of transfection efficiency; compared liposome-DNA complexes and electroporation assisted DNA delivery using two different sets of electrical parameters.

## Methods

### Animals and tumors

All animal experiments were carried out in accordance with the UK animals (Scientific Procedures) Act 1986, following the UKCCCR guidelines and with approval from the local Ethical Review Committee of the Gray Cancer Institute.

Early generations of the P22 transplanted rat carcinosarcoma were used in experiments. Donor tumors were grown subcutaneously in the left flank of 8–10 week old male BD9 rats [18].

### Surgery

Surgery was carried out under general anesthesia using intraperitoneal injection of fluanisone (10 mg/kg), fentanyl citrate (0.315 mg/kg) ('Hypnorm', Janssen Animal Health, UK) and midazolam (2 mg/kg) ('Hypnovel', Roche Products Ltd., UK) as described previously [18]. Briefly, animals were kept warm using heating pads throughout the surgical procedure and aseptic technique was used throughout. Window chambers, consisting of a double-sided aluminum frame, holding two parallel glass windows approximately 200 μm apart, were surgically implanted into the dorsal skin of male BD9 rats, weighing approximately 200 g. Surgery involved removal of the epidermal and dermal layers of both skin layers of a dorsal skin flap, except for the deepest fascia layer on each side, and then securing the two sides of the chamber to the skin using stainless steel screws and sutures. The two fascia layers moved freely between the two glass windows, following these procedures. Early generation subcutaneous transplants of the P22 rat carcinosarcoma were used as donor tumors, when they reached approximately 0.5 cm in diameter. A tumor fragment (~0.5 mm diameter) was placed onto one of the fascia layers within the window chamber on the day of surgery. Animals were given an intraperitoneal injection of a few milliliters dextrose-saline, immediately following surgery and kept on a warmed pad until recovery from anesthesia. Subsequently, animals were kept in a warm room (30–34°C) until the day of experiment. Three animals were used for each experimental group.

### Study design

Transfection was carried out 7 to 14 days following surgery, when tumors measured 3–4 mm in diameter, using a plasmid encoding the green fluorescent protein (GFP; p-EGFP-N1, Clontech, Basingstoke, UK). Animals were anaesthetized with Hypnorm and midazolam. The window on the tumor side was removed and 40 μg of DNA alone or encapsulated in lipofectin (30 μl; Life Technologies, Paisley, UK) vesicles was carefully placed on the tumor surface in a total volume of 75 μl in phosphate buffered solution [5]. One minute thereafter, electroporation was carried out by application of 8 square electric pulses generated by an electroporator (built in-house). Pulses were delivered by two flat, parallel stainless steel electrodes (two stainless steel strips: length 15 mm, width 4 mm with rounded corners) 3 mm apart that were placed at the diametrically opposed edges of the tumor. Two different electroporation protocols were used: **EP1 **– amplitude 180 V (voltage/distance ratio 600 V/cm); pulse length 5 ms; repetition frequency 1 Hz and **EP2 **– amplitude 390 V (voltage/distance ratio 1300 V/cm); pulse length 0.1 ms; repetition frequency 1 Hz. Immediately after the procedure, the glass window was replaced and GFP fluorescence monitored at selected time points for 6 days.

### Intravital microscopy

Intravital microscopy was carried out using an inverted Nikon Diaphot 200 fluorescence microscope, with a stage modified in-house for holding rats. Animals were anaesthetized with Hypnorm and midazolam and placed on the stage, in such a way that the window chamber was located centrally above the objectives using location screws. Rectal temperature was maintained between 34–37°C throughout the experiment, using a thermostatically controlled heating pad beneath the rat and an infrared overhead lamp for maintenance of tumor temperature.

Tumor preparations were alternatively viewed at each time point under transmitted visible light for measurement of tumor diameter and under fluorescence epi-illumination using a 100 W mercury arc lamp, for visualization of GFP fluorescence (excitation filter of 450–490 nm, emission filter of 520 nm) using x1.6, x4, x10 and x20 objective. Prior to transfection, two different regions of interest (ROI) using x10 objective were selected, one in the center of the tumor and one in the tumor periphery. Tumor preparations were monitored at each time point for 15 s using transmitted or epi-illumination.

### Data analysis

Observations were recorded in digital format, using a Sony DSR-30P digital videocassette recorder, for off-line analysis. Multiple frames (typically 10) were captured onto computer and the images averaged for the analysis of the fluorescence intensity using the Visilog Image Processing package (Noesis, France). Increase in fluorescence intensity in the tumors was determined by subtracting the values of fluorescence intensity of non-transfected granulation tissue from the values of tumors and normalizing them to the values of non-transfected tissue. The calculations were performed for each animal at all time points.

### Statistical analysis

Statistical analysis was carried out using SigmaStat Statistical software (Systat Software GmbH, Erkrath, Germany). Data were tested for normality using Kolmogorov-Smirnov test and differences between the groups were tested for significance using Holm-Sidak method, after one-way repeated measures analysis of variance was performed. A value of p < 0.05 for the comparisons was considered to represent a significant difference between groups.

## Results and discussion

**Figure 1:**
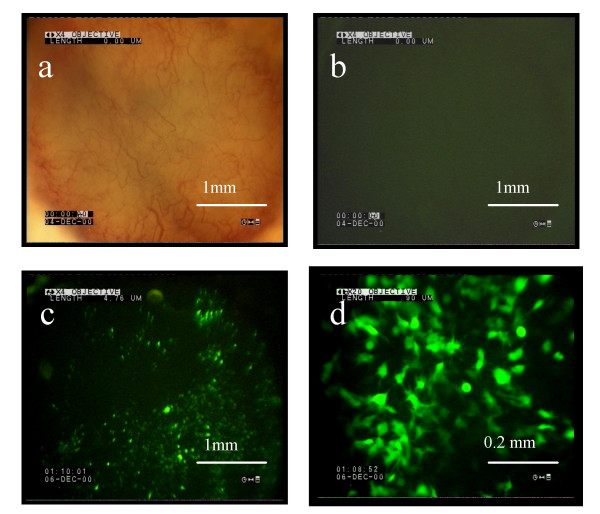
**Expression of GFP following electroporation-assisted gene delivery using EP1 **GFP expression was monitored in the rat P22 tumor growing in rat dorsal flap window chamber following electroporation-assisted gene delivery using EP1 (8 electric pulses, 600 V/cm, 5 ms, 1 Hz). *a. *image taken under visible light condition on day 0 (x4 objective), *b. *image taken under fluorescence epi-illumination on day 0 before administration of DNA and electroporation (x4 objective), *c. *image taken under fluorescence epi-illumination on day 2 (x4 objective); *d. *image taken under fluorescence epi-illumination on day 2 (x20 objective).

Three different types of non-viral transfection methods were compared: DNA alone, liposome-DNA complex and electroporation-assisted DNA delivery, using two different sets of electric pulse parameters. Transfection of GFP was monitored using intravital microscopy on syngeneic P22 rat carcinosarcoma tumors growing in the dorsal skin flap window chamber of BD9 rats (Figure 1). The transfection efficiency was evaluated by monitoring real time spatial distribution and time dependence of GFP fluorescence. Among the tested transfection methods, electroporation-assisted gene delivery was the most effective method for transfection of tumors growing in the window chamber. Two different sets of electric pulse parameters were tested; EP2 resulted in up to 17%, while EP1 in up to 22% increase in fluorescence intensity in the tumors. EP2 has previously been proven to be effective in electrochemotherapy of accessible cutaneous tumors in patients with histologically different types of tumors [6,7], and also for electrogenetherapy [8]. On the other hand, EP1 electric pulses have already been shown to be effective for gene delivery of solid tumors of different histology and origin (rat, mouse and human) [5]. Transfection efficiency using either DNA alone or liposome-DNA complex was lower (7% and 12%, respectively) compared to electroporation-assisted gene delivery.

**Figure 2:**
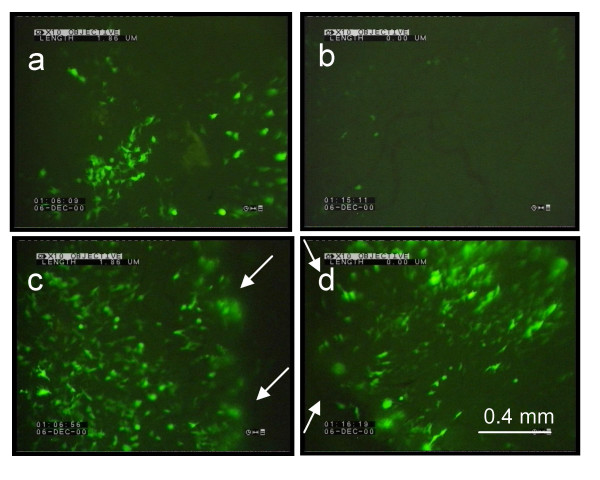
**Comparison of transfection efficiency between tumour center and periphery following two different electroporation protocols **GFP expression in rat P22 tumor growing in rat dorsal flap window chamber 2 days after two different electroporation conditions: EP1: 8 electric pulses, 600 V/cm, 5 ms, 1 Hz (*a*, *c*); EP2: 8 electric pulses, 1300 V/cm, 0.1 ms, 1 Hz (*b*, *d*). Comparison of transfection efficiency in the ROI's of tumor center (*a*, *b*) and periphery (*c*, *d*) (x10 objective). Arrows: edge of the tumor.

Spatial distribution of transfected cells differed between the two sets of electric pulses parameters. When using EP1 electric pulse parameters, cells expressing GFP were more spread out through the whole tumor, compared to EP2 conditions, where cells expressing GFP were limited to the areas that were close to the positioning of the electrodes (Figure 2). Electroporation of the cell membrane is a physical phenomenon that occurs above a certain threshold of induced transmembrane potential and is dependent on electric pulse parameters such as pulse length, pulse amplitude, number and pulses sequence [4]. According to the current knowledge at a single cell level, DNA electro-transfer is a process involving attachment of DNA to the electropermeabilized side of the cell facing the cathode, aggregation of DNA and its translocation to the cytoplasmic side of the cell [19]. Therefore, in the case of EP2, the conditions suitable for DNA electro-transfer (above the threshold level) *in vivo *appeared to be obtained only in the vicinity of the electrodes, whereas in the case of EP1 a larger area of the tumor was prone to DNA transfer. Effectiveness of electroporation-assisted gene delivery was also evident by lack of transfection efficiency in the granular tissue surrounding the tumor that was not exposed to electric pulses, but to DNA only (Figure 2). In addition, more homogenous distribution of transfection efficiency using EP1 conditions compared to EP2 conditions could be due to the electrophoresis of the DNA caused by longer duration of the EP1 pulses.

**Figure 3:**
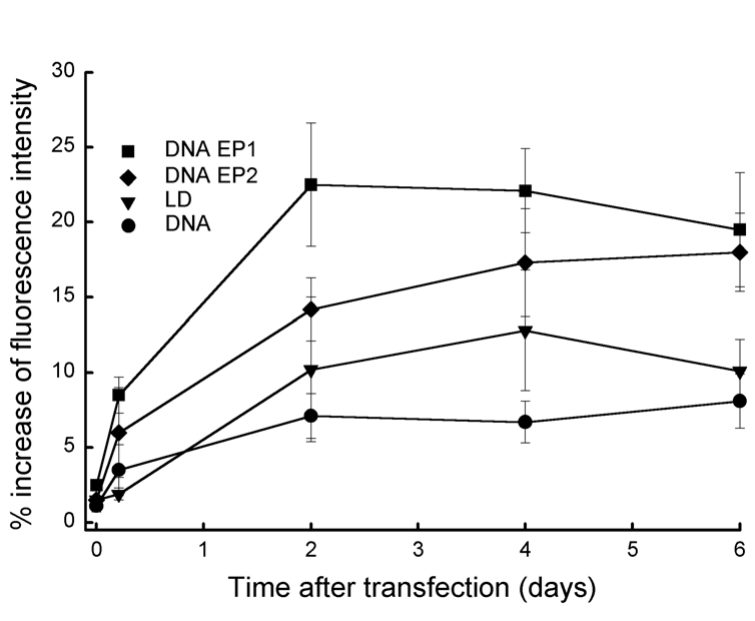
**Time dependence of transfection efficiency comparing different transfer methods **DNA or liposome-DNA complexes were administered to the top of the tumor growing in window chamber. Electric pulses were applied to the tumor immediately after the addition of DNA. Two sets of electric pulse parameters were used: EP1 (8 electric pulses, 600 V/cm, 5 ms, 1 Hz) and EP2 (8 electric pulses, 1300 V/cm, 0.1 ms, 1 Hz). Average of three animals/group +/- standard error of the mean.

**Figure 4:**
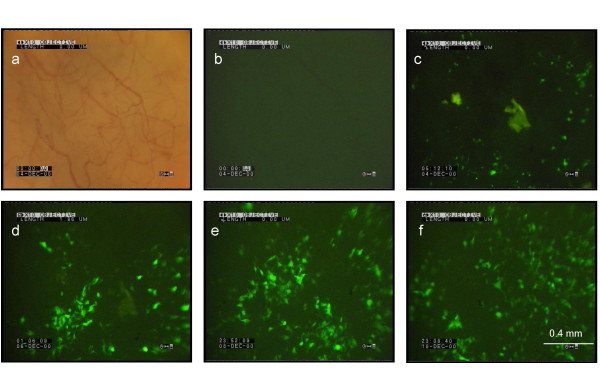
**Representative images of time related transfection efficiency of electroporation-assisted gene delivery using EP1 conditions **Example images taken under visible light condition (*a*) and under fluorescence epi-illumination (*b*) on day 0 before administration of DNA and electroporation. Example images of transfection efficiency in tumors at 5 h (*c*), 2 (*d*), 4 (*e*) and 6 days post-transfection (*f*). (x10 objective).

Functional GFP was formed within 5 h after transfection regardless of the transfection method used (Figures 3, 4). Detectable green fluorescence is the end result of a series of events, including transfer of the DNA encoding GFP into the cell, evasion of intracellular nucleases, transfer to the nucleus, transcription and translation, and finally, folding of the protein into a functional protein's fluorophore. Topical administration of DNA to the tumors in the window chamber (40 μg of DNA) resulted in very low transfection efficiency, the increase in fluorescence intensity in the tumors was up to 7% at day 2 post-transfection, and remained at this level up to day 6. Administration of liposome-DNA complexes resulted in increased transfection efficiency in the tumors compared to DNA alone (P = 0.032). The increase was up to 12% with the similar level of GFP expression on day 6 post-transfection as after the administration of DNA only (Figure 3). Similar results were obtained in our previous study on dense cell suspensions and solid subcutaneous P22 tumors where liposome-DNA complexes resulted in significantly higher GFP transfection efficiency compared to DNA injection only [5]. These results are in accordance with several preclinical and clinical studies demonstrating efficient gene-transfer to solid tumors using liposome-DNA complexes [20,21]. The highest increase in fluorescence intensity was obtained with electroporation-assisted gene delivery using EP1 in the present study. The fluorescence intensity increased to 22% at day 2 post-transfection and then remained at this level over the observation period of 6 days. Electroporation assisted gene delivery with EP2 was less effective than with EP1, although it was better than liposome-DNA complex alone. These results are in accordance with our previous study, where electroporation assisted gene delivery using either of the electric pulse parameters yielded higher transfection efficiency compared to lipofectin-enhanced method. The transfection efficiencies that were obtained in the present study were much higher compared to the transfection efficiency that we obtained in our previous study on P22 solid tumors growing subcutaneously in SCID mice [5]. The possible reason for this is the absence of the skin overlying the tumors in the window chamber. In subcutaneously growing tumors, the presence of the stratum corneum of the skin causes the electric field intensity to drop substantially, especially in the centre of the tumor, compared to the electric field intensity in the skin [22,23]. The absence of this effect in the window tumors may account for the increased transfection efficiency observed in the current study, as predicted from theoretical analyses [22,23].

It is worth noting that the fluorescence level remained approximately constant throughout the observation period for all transfection methods. This means that, as the tumors grew during the 6-day observation period (tumor diameter increased approximately 2-fold), plasmid DNA appeared to be present in the progeny cells. In addition, intravital microscopy demonstrated that application of electric pulses to the tumors did not induce major cell damage as no effect on the tumor growth was observed compared to untreated controls. Six days post transfection there was no difference in increase in tumor diameter between the tumors that were transfected with DNA alone (2.2 fold), liposome-DNA complexes (2.3 fold), and electroporation-assisted DNA delivery using EP1 (2.4 fold) or EP2 (2.2 fold).

## Conclusions

In conclusion, this study showed that intravital microscopy is useful for monitoring the spatial and temporal efficacy of electroporation methods for gene transfection in animal tumor models. As such, it will be valuable for the evaluation of new methods of optimizing gene delivery. Electroporation-assisted gene delivery using EP1 was found to result in early onset and homogenous distribution of gene expression in the P22 tumor model. Further improvement in transfection efficiency may be gained by optimizing electric pulse parameters.

## Competing interests

The author(s) declare that they have no competing interests.

## Authors' contributions

MC conceived the study, participated in its design and experiments, and drafted the manuscript. IW participated in design of the study and experiments. GUD participated in design of the study and critically revised the draft. GMT participated in design of the study and coordination, and critically revised the draft. GS helped to draft the manuscript and participated in analysis and interpretation of the data.

## Pre-publication history

The pre-publication history for this paper can be accessed here:
